# Laser Fabrication of Miniature Internal Thread in Glass Substrate

**DOI:** 10.3390/mi8020048

**Published:** 2017-02-08

**Authors:** Hiroyuki Degawa, Noriaki Urano, Shigeki Matsuo

**Affiliations:** Department of Mechanical Engineering, Shibaura Institute of Technology, Tokyo 135-8548, Japan; aa12073@shibaura-it.ac.jp (H.D.); aa12013@shibaura-it.ac.jp (N.U.)

**Keywords:** subnanosecond laser, miniature thread, glass, water-assisted laser drilling

## Abstract

Miniature internal threads (tapped holes) of S0.5 were fabricated in a glass substrate. Water-assisted laser drilling was applied for fabrication of the threads of S0.5 standard using a subnanosecond laser as a light source. The landscape of the inner surface of the threads was measured by a laser microscope, and showed reasonable agreement with the desired standard. As a proof of concept, a commercial external screw was fitted to the fabricated internal thread.

## 1. Introduction

Miniaturizing mechanical, microfluidic, and electronic devices is a trend of modern technology. In particular, lab-on-a-chip approaches aim at scaling entire laboratories down into small chips, often made in glass. The needs for jointing techniques suitable for assembling small parts are increasing. One such jointing technique is fastening by miniaturized screws. There is a standard for miniature screw threads defined by the International Organization for Standardization (ISO); ISO1501.

For the fabrication of a miniature internal thread, a rolling tap is often used. It is, however, impossible to apply the rolling method to brittle materials such as glass, because brittle materials do not undergo plastic deformation. A cutting tap is also not applicable to brittle materials.

In the present study, we demonstrate the capability of laser processing to manufacture miniature internal threads in glass substrates. Laser processing has been applied to the cutting and drilling of a wide range of materials, including glass. For drilling, lasers are usually used to fabricate straight holes. However, for the fabrication of an internal thread (tapped hole), ridges and grooves need to be fabricated in the inner surface of the hole; this is a challenging task for laser processing.

We evaluated whether water-assisted laser drilling [1,2,3,4] is the most adequate processing method for this task, thus adopted in the present study. Water-assisted laser drilling is a technique for subtractive processing by laser ablation; during the process, the sample is immersed in water (or other liquid). The ablated material is then dispersed in water; thus, re-attachment of the ablated material is suppressed, and the ablated material is efficiently ejected from the hole. This technique has been applied to fabricate arbitrary-shape three-dimensional micro hollow structures such as microchannels perpendicular to the direction of the laser beam. We apply this technique to a machine element: miniature internal thread.

In the present study, we used a subnanosecond laser as the light source for water-assisted laser drilling, while a femtosecond (fs) laser is often used for this application.

## 2. Materials and Methods

The light source used was a subnanosecond Nd:YAG laser (PNP-M08010, Teem Photonics, Meylan, France), which emits 0.5 ns, 90 μJ pulses at 1064 nm with a maximum repetition rate of 1 kHz; a femtosecond (fs) laser is often adopted for this technique. Second harmonic (SH) was generated using a ${\mathrm{LiB}}_{3}{\text{O}}_{5}$ crystal with pulse energy up to ∼40 μJ, and used for experiments. The SH pulses were led to an optical microscope (IX-70, Olympus, Tokyo, Japan) and focused by a near-infrared objective lens (PLN20x, Olympus, numerical aperture of 0.40, working distance of 1.2 mm). The pulse energy was measured by a pyroelectric energy sensor (PE10-C, Ophir Optronics, Jerusalem, Israel) before the entrance of the microscope.

**Figure 1:**
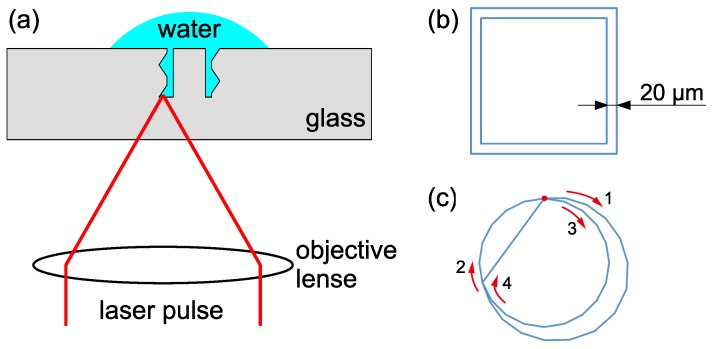
(**a**) Schematic view of water-assisted laser drilling; (**b**) The trajectory of the laser focus at each *z*-plane for a hole with a square cross-section; (**c**) The trajectory of the laser focus at each *z*-plane for an internal thread. The numbers indicate the order of the movement.

The substrate in which miniature internal thread was fabricated was a glass slide (S1111, Matsunami Glass, Kishiwada, Japan) with a thickness of ∼1.0 mm. The glass slide was translated using a three-dimensional motorized stage (combination of ALS-6012-G1M (*x* and *y*) and ALV-600B-H1M (*z*), Chuo Precision Industrial, Tokyo, Japan). The stage was controlled with a simplified language supplied with the stage (for square holes) or a home-made LabVIEW program (for internal threads). The laser irradiation started from the rear (upper) surface, then moved to the front (lower) side; accordingly, the laser beam did not pass the already-processed region. For the fabrication of hollow structures, the substrate was translated so that the trajectory of the laser focus moved on the surface layers of the hollow region—instead of moving the whole hollow region—in order to reduce processing time. This is schematically shown in Figure 1a.

Practically, the trajectory of the laser focus was moved on two layers: the outer-most layer, and the slightly inside layer. In addition to the material removal by laser ablation, the material between the two layers was broken into fragments and dispersed into water. This promoted the infiltration of water, and reduced the formation of cracks. The detail of the trajectory will be described later.

The fabricated threads were observed by optical microscope. In addition, the landscape of the inner surface of the threads was measured by a laser-scanning microscope (OLS4000, Olympus) after cleaving the substrate. To cleave the substrate exactly along the center of the fabricated thread, a laser-induced internal transformation method [5] was applied.

## 3. Results and Discussion

**Figure 2:**
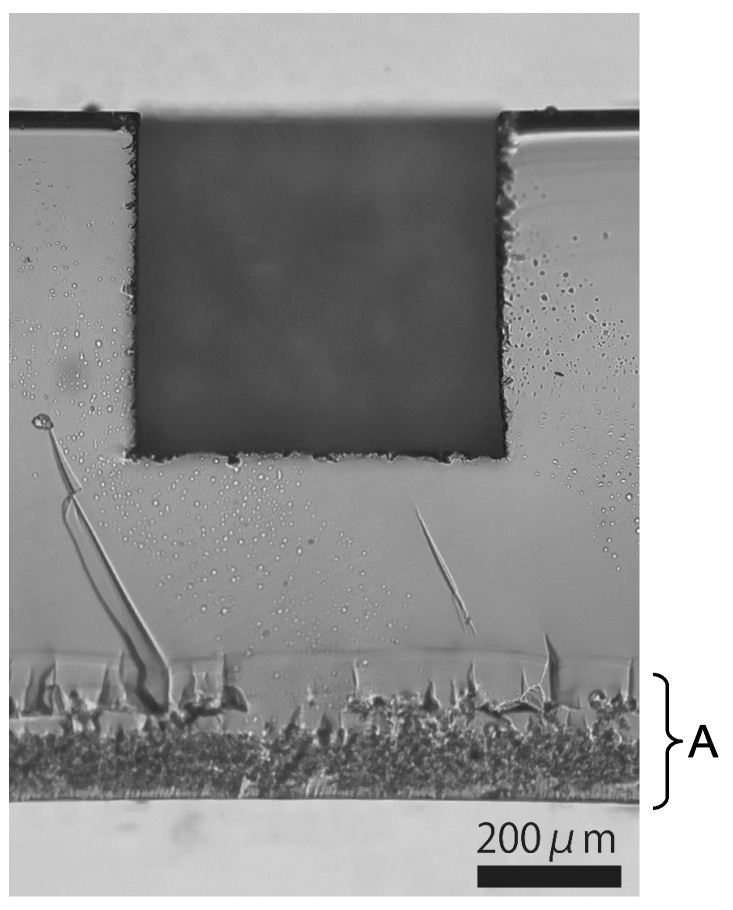
Sectional view of a fabricated hole with square cross-section. “A” indicates the region where laser-induced internal transformation was applied for cleaving.

As a preliminary trial, we fabricated a non-through hole with a square cross-section of side length about 500 μm. In this case, in addition to the most-outer layer, a 20 μm-inside-layer was irradiated for each *z*-plane, as shown in Figure 1b. The pulse energy was 20 μJ, the repetition rate was 1 kHz, and the translation speed of the sample was 1 mm/s. These parameters were selected for fast and repeatable fabrication, and to minimize the formation of cracks. Figure 2 shows the sectional view. This image shows the ability of this technique to drill inside glass substrate. The depth of this non-through hole was about 474 μm, while the sample was translated by 300 μm. The ratio 1.58 is slightly larger than the refractive index of the sample, 1.524 (at 546.1 nm) [6]. This is reasonable when the difference in focus position due to spherical aberration arising at the air–glass interface was taken into account [7]. We adopted this value for defining the pitch of thread.

**Figure 3:**
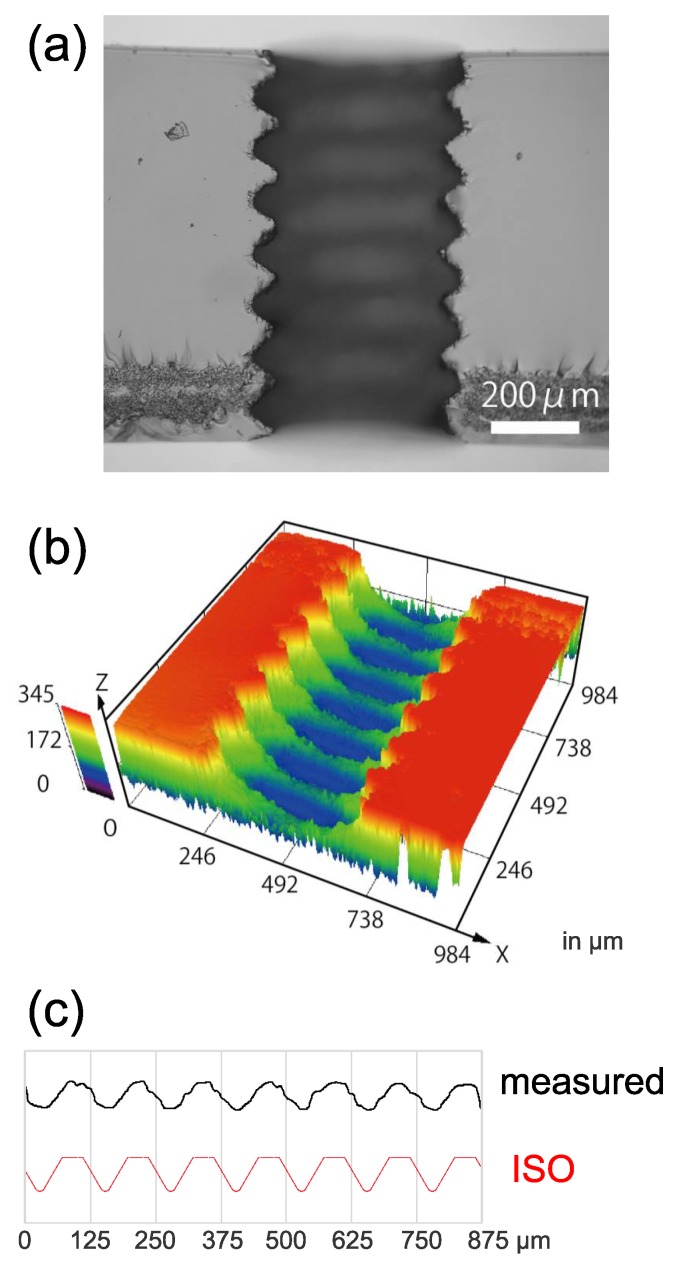
Internal surface of fabricated threaded hole after cleaving. (**a**) Optical microscopy image; (**b**) Laser-scanning microscopy image (oblique view), the unit is μm; (**c**) Comparison of cross-sectional profile between fabricated thread and ISO1501. The vertical scale is the same as the horizontal scale.

Next, we fabricated a miniature internal thread of S0.5 (major diameter of 0.5 mm) through the glass substrate. The pulse energy, repetition rate, and translation speed were the same as those for the hole with a square cross-section. The trajectory of the laser focus was as shown in Figure 1c. The focus was at first moved along the outer-most layer, which consists of the groove (1); and the ridge (2); then, it was moved along the minor diameter circle (3); and finally moved to the original position (4). Fabrication of one thread took about 32 min. Figure 3 shows the images of the inner surface of the fabricated thread after cleaving. Figure 3a is the sectional view. We can see a spiral pattern of the thread. Figure 3b is a pseudo-color oblique view measured with laser-scanning microscopy. This image clearly shows that ridges and grooves were recorded on the inner surface. The cross-sectional profile and its comparison to that of the ISO is shown in Figure 3c. The pitch of the fabricated thread reasonably agreed with the ISO standard (0.125 mm), while some chipping (small cracks) was observed.

**Figure 4:**
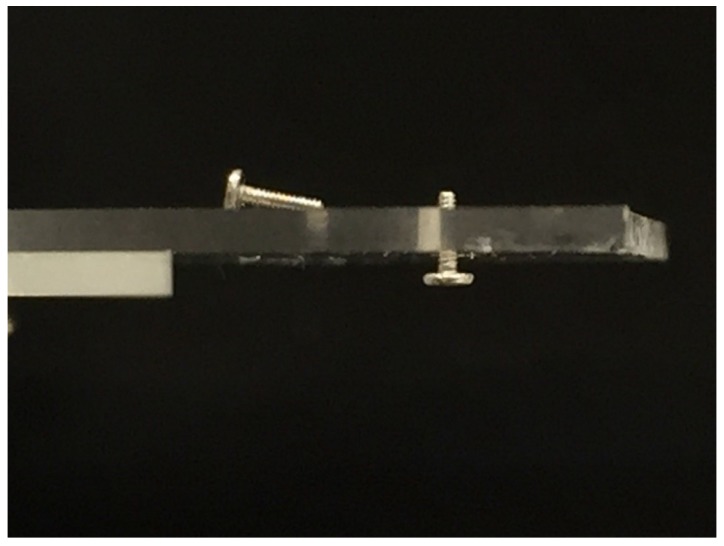
Joint of a S0.5 male screw (nominal length of 2 mm) to the fabricated internal thread.

In order to examine the functionality of the fabricated internal thread, a commercial S0.5 male screw (Matsumoto Industry, Abiko, Japan) was fitted to it. The photo is shown in Figure 4. As seen, the male screw was well fitted through the hole, and remained in the hole against the force of gravity.

In the present study, S0.5 internal threads were fabricated with a depth of about 1 mm. Smaller threads are also defined by ISO1501 down to S0.3. In downsizing the threads with the present technique, the expectable problems are chipping and spatial resolution. S0.3 might be attainable, but further downsizing—with subnanosecond laser processing—would be almost impossible because of the problems mentioned above. To address this issue, a shorter pulse laser—such as a femtosecond laser—could be deployed.

On the other hand, for the fabrication of a deeper internal thread, the expectable problem is insufficient working distance of the objective lens. In addition, suppression of spherical aberration in the whole processing range will also be a serious problem. The use of a wave-front correction device such as a spatial light modulator [8] will play an important role for aberration compensation.

Another three-dimensional micro removal processing technique—modification by focused fs pulses followed by chemical etching—has been developed with fs lasers [9,10,11,12]. Gottmann et al. cut out a micro gear from a fused silica substrate [13]. They also produced a free-rotating gear that is already mounted on its axis [13]. This technique will also be applicable for the fabrication of miniature internal threads. In this case, it is impossible to completely avoid the etching of non-modified region [14,15]. This could be a problem for high-precision fabrication of the threads. The present technique is free from this problem. Additionally, the advantage of the present technique is its simpler process and the lack of need for chemical reagents.

## 4. Conclusions

A S0.5 miniature internal thread was fabricated in glass substrate by a laser technique—water-assisted laser drilling—with a subnanosecond laser as a light source. The inner surface of the thread was observed after cleaving the glass substrate along the center of the thread. Ridges and grooves were observed, and the cross-sectional profile reasonably agreed with that of ISO standard. The functionality of the thread was demonstrated by fitting a commercial male screw. This technique could be applicable for other types of hollow structures, such as microchannels.
